# mRNA translational dynamics mediates totipotent-like reprogramming

**DOI:** 10.1016/j.jbc.2026.113464

**Published:** 2026-08-19

**Authors:** Lingci Huang, Jun Zhang, Xinwei Wu, Haotian Zhang, Wanting Cai, Xiaoyan Sun, Yuanhui Mao, Jun Zhou, Xiao-Min Liu

**Affiliations:** 1 School of Life Science and Technology, China Pharmaceutical University, Nanjing, Jiangsu, China; 2 Department of Neurology of The Second Affiliated Hospital & Liangzhu Laboratory, Zhejiang University School of Medicine, Hangzhou, China

**Keywords:** 2CLCs, mitochondrial translation, mRNA translation, totipotent, cell reprogramming

## Abstract

Embryonic stem cells (ESCs) are characterized by their dual capacity for self-renewal and differentiation into all cell types of the embryonic lineage. A subpopulation known as 2-cell-like cells (2CLCs), which recapitulate key molecular and metabolic features of totipotent 2-cell blastomeres, has been identified within cultured mouse ESC populations. While transcriptional regulation, epigenetic modifications, and chromatin reorganization are known to be critical for the reprogramming of pluripotent ESCs into a totipotent-like state, the role of translational control in this process remains poorly understood. Using an inducible 2CLC model, we performed transcriptome-wide profiling of mRNA translation and found that global translation efficiency dynamically decreases during the early phase of totipotent-like reprogramming, correlating with reduced TORC1 signaling and translation initiation. In the later phase, although overall mitochondrial mass declines, mitochondrial translation is selectively upregulated and exhibits high translational efficiency. Importantly, pharmacological inhibition of mitochondrial translation suppressed the expression of canonical 2-cell transcripts and impaired the transition from ESCs to 2CLCs. Together, these results demonstrate the coordinated regulation of both cytosolic and mitochondrial translation during totipotent-like reprogramming, offering a new perspective for understanding cell fate determination.

Embryonic stem cells (ESCs), derived from the inner cell mass, possess the capacity to differentiate into all cell types of embryonic lineage rather than extra-embryonic tissues (1). Although they are considered pluripotent, ESCs maintained in culture display notable heterogeneity. Among them, a small fraction (typically less than 1%) spontaneously enters a state that closely resembles the totipotent 2-cell embryo. These cells are known as 2-cell-like cells (2CLCs) (2, 3). They transiently activate a transcriptional program characteristic of the 2C stage, including the expression of specific markers such as *Dux*, *Zscan4* and *MERVL*, and demonstrate the ability to contribute to both embryonic and extraembryonic tissues in chimeric assays (4, 5, 6, 7). This conversion of pluripotent ESCs to 2CLCs, termed totipotent-like reprogramming, provides a valuable and controllable *in vitro* model for studying the mechanisms that establish and regulate cellular totipotency, thereby offering critical insights into the earliest events of mammalian development.

Totipotent-like reprogramming is primarily orchestrated by a defined transcriptional program. At its core is the double homeobox (DUX) protein, a master transcription factor that directly binds to and activates 2C-specific genes and retrotransposons, including *Zscan4c* and *MERVL*, thereby initiating the reprogramming process (8, 9, 10, 11). This DUX-centric program is supported and amplified by other regulators, such as TRIM66, NELFA, p53 and DPPA2/4, which promote the transition to a totipotent-like state primarily through activating or stabilizing the expression of DUX itself (12, 13, 14, 15, 16, 17). Beyond transcriptional activation, epigenetic remodeling is equally critical for this cell fate transition. During the reprogramming from ESCs to a 2C-like state, cells undergo genome-wide DNA demethylation. This process erases the pluripotent epigenetic signature and licenses the expression of 2C-specific genes and retrotransposons like *MERVL*. Notably, this demethylation pattern is, at least in part, orchestrated by the ZSCAN4-DNMT1 axis (10, 18, 19). Concurrently, dynamic histone modifications are also crucial for establishing totipotency. For example, promoters of highly upregulated 2C genes like *Zscan4* and *Dux* gain *de novo* H3K4me3 marks in 2CLCs (20). Specific histone lysine demethylases, including KDM5B and KDM1A, have also been implicated in facilitating this cell state transition (4, 21). Beyond DNA and histone modifications, RNA epitranscriptomic marks such as *N*^6^-methyladenosine (m^6^A) further contribute to acquiring a totipotent-like state (22, 23, 24, 25). Thus, the coordinated action of transcription factors, epigenetic remodeling, and RNA modifications collectively establishes a permissive molecular environment that enables totipotent-like reprogramming.

Translation represents a tightly regulated and dynamic layer of gene expression, enabling rapid post-transcriptional control of protein abundance independent of mRNA levels. This regulatory mechanism is particularly critical during dramatic cell state transitions, such as early embryogenesis, stem cell differentiation, and stress or immune responses, where the proteome must be swiftly and decisively remodeled (26, 27, 28, 29). While transcriptional and epigenetic regulators of totipotent-like reprogramming are well established, the potential contribution of translational regulation to the establishment of a totipotent-like state remains largely unknown. Therefore, a systematic analysis of translatome dynamics during this process is essential to achieve a comprehensive understanding of the molecular principles governing totipotency.

To address this gap, we performed ribosome profiling (Ribo-seq) coupled with RNA sequencing to capture the dynamic translational landscape during totipotent-like reprogramming of ESCs to 2CLCs. Our integrated analysis revealed a profound yet transient suppression of global translation, orchestrated through multiple signaling pathways. Intriguingly, within this context of broad translational repression, we discovered a selective and essential upregulation of mitochondrial translation, which is indispensable for the acquisition of the totipotent-like state.

## Results

### Repression of global mRNA translation during totipotent-like reprogramming

To elucidate translational dynamics during totipotent-like reprogramming, we utilized stable 2C-tdTomato transgenic mouse ESCs. This cell line harbors both a doxycycline (Dox)-inducible *Dux* transgene and a *MERVL* promoter-driven tdTomato reporter, as previously described (10). We first conducted a time-course experiment to identify the optimal time points capturing the transition from pluripotent ESCs to totipotent-like 2CLCs. Flow cytometry assay revealed a gradual increase in the population of tdTomato^+^ cells following Dox induction, reaching a peak at 24 h and showing a moderate decline by 36 h (Fig. S1, *A* and *B*). Based on this kinetics, we selected 0 h (preinduction), 12 h (mid-transition), and 24 h (peak transition) for subsequent multi-omics profiling to comprehensively characterize the gene expression changes underlying this cell fate conversion. Consistent with the reprogramming trajectory, analysis of canonical markers confirmed a time-dependent upregulation of totipotent genes (*Dux*, *Zscan4*, *MERVL*) and a concomitant downregulation of pluripotent markers, such as *Nanog*, *Pou5f1*, *Sox2* (Fig. S1, *C* and *D*).

**Figure 1:**
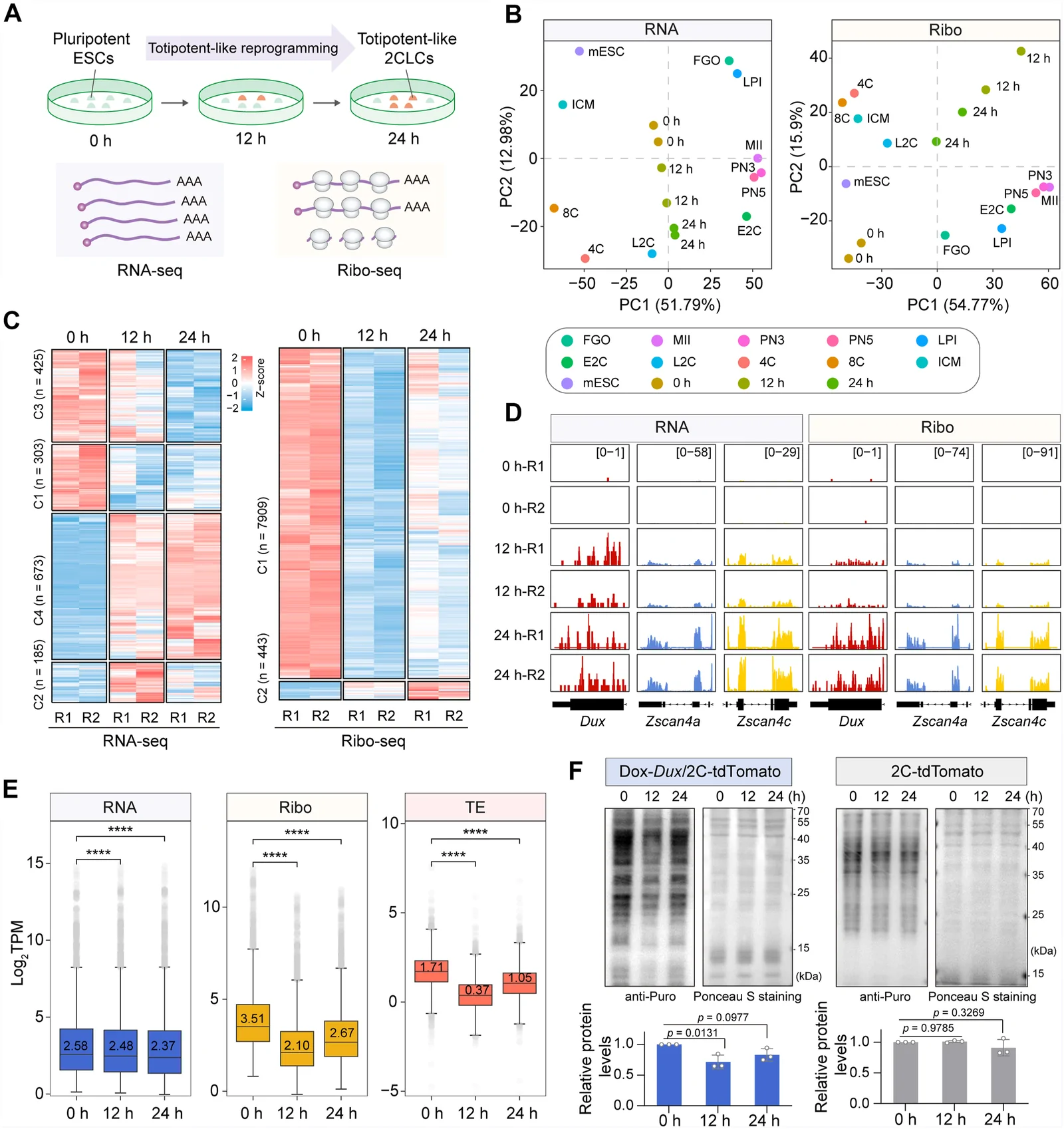
**Global mRNA translation is suppressed during totipotent-like reprogramming.***A,* schematic of the experimental timeline and multi-omics strategy. ESCs were collected at 0, 12, and 24 h post-induction of totipotent-like reprogramming for parallel RNA-seq and Ribo-seq analyses. *B,* principal component analysis of transcriptomes *(left)* and translatomes *(right)* for this study with mouse oocytes and early embryos, including FGO (fully grown oocytes), LPI (late prometaphase I oocytes), MII (metaphase II oocytes), PN3 (early one-cell stage), PN5 (late one-cell stage), E2C (early two-cell stage), L2C (late two-cell stage), 4C (four-cell stage), 8C (eight-cell stage), ICM (inner cell mass of blastocyst), and ESC (mouse embryonic stem cells). *C,* heatmaps showing the expression dynamics of genes clustered by their mRNA abundance *(left)* and ribosome density *(right)* across indicated timepoints during totipotent-like reprogramming. *D,* genome browser view of representative regions for canonical 2-cell-stage marker genes (*e.g.*, *Dux*, *Zscan4a*, *Zscan4c*) in cells across the three timepoints. *E,* boxplots showing the distribution of log_2_TPM values for mRNA abundance (RNA-seq), ribosome density (Ribo-seq), and TE of all genes at different timepoints. Each *box* represents the interquartile range with the median indicated. Statistical significance was determined by the Wilcoxon test (∗∗∗∗*p* < 0.0001). *F,* global protein synthesis during totipotent-like reprogramming was measured by the SUnSET assay. *Top left*: Representative blot showing puromycin incorporation in Dox-inducible *Dux* double-reporter ESCs (Dox-*Dux*/2C-tdTomato) at 0 h, 12 h, and 24 h post-Dox induction. Top right: Representative blot showing puromycin incorporation in *MERVL*-tdTomato single-reporter ESCs lacking the Dox-inducible *Dux* system (2C-tdTomato) treated with Dox (2 μg/ml) at 0 h, 12 h, and 24 h as a negative control. Cells were labeled with puromycin (10 μg/ml, 15 min) prior to harvest. Ponceau S staining is shown as a loading control. *Bottom*: Quantification of puromycin incorporation signals for Dox-*Dux*/2C-tdTomato reporter cells *(left)* and 2C-tdTomato reporter cells *(right)*. Signal intensities were normalized to the 0 h control. Data are presented as mean ± S.D. from independent biological replicates (n = 3), exact *p* values were included, one-way ANOVA with Dunnett's multiple comparisons test. ESC, embryonic stem cell; TE, translational efficiency.

We subsequently captured the translatome and transcriptome changes using Ribo-seq and RNA-seq in parallel during totipotent-like reprogramming at 0, 12, and 24 h (Fig. 1*A*). Correlation analysis indicated high reproducibility between the independent replicates (Fig. S2*A*). Consistent with the expected size of ribosome-protected fragments (RPFs) in mammalian cells, the size distribution of RPFs of Ribo-seq showed a predominant peak at approximately 30 nucleotides (Fig. S2*B*). Moreover, the majority of RPFs (>85%) were uniquely mapped to protein-coding sequences (CDSs) (Fig. S2*C*), indicating the high quality of the Ribo-seq datasets.

To systematically quantify the dynamic changes in both mRNA abundance and ribosome density, we performed principal component analysis. This analysis revealed a pronounced separation of the 12-h and 24-h samples from the 0-h controls (Fig. S3*A*), revealing extensive transcriptional and translational remodeling. To place these dynamics within a physiological context, we integrated our datasets with published transcriptome and translatome profiles from mouse early embryonic development. This comparative analysis showed that 0-h samples clustered with reference pluripotent ESCs, whereas 24-h samples aligned closely with the late 2-cell stage (Fig. 1*B*). These patterns suggest that the reprogramming process recapitulates key mechanistic aspects of totipotency acquisition.

We next performed time-series clustering to characterize global expression dynamics of the transcriptome and translatome during reprogramming. Transcriptomic profiles partitioned into four distinct dynamic clusters. Genes in cluster 1, which were downregulated at both 12 h and 24 h, were enriched for biological processes related to stem cell population maintenance and embryonic development (Fig. S3*B*), whereas upregulated genes (cluster 4) showed enrichment for functions in RNA transcription and protein homeostasis (Fig. S3*C*). In contrast, translatome profiles segregated into only two major clusters (Fig. 1*C*). This pronounced difference in regulatory complexity suggests a substantial decoupling of transcriptional and translational control during the pluripotent-to-totipotent-like transition. As classic 2C-specific genes, *Dux* and *Zscan4*, exhibited coordinated progressive increases in both mRNA abundance and ribosome occupancy from 0 to 24 h post-induction (Fig. 1*D*), further indicating the successful activation of a totipotent-like transcriptional and translational program.

To further quantify the differential remodeling between these two regulatory layers, we compared the genome-wide dynamics of mRNA abundance and ribosome occupancy. Principal component analysis revealed tighter clustering of RNA-seq samples across time points, whereas Ribo-seq samples displayed substantially greater dispersion (Fig. S4*A*). The distribution of RNA log_2_fold change remained centered on 0 at both time points, whereas RPF log_2_fold change distribution shifted leftward at 12 h and largely recovered by 24 h. Furthermore, at 12 h, Ribo-seq revealed significant changes in 49.6% of genes, *versus* only 1.7% for RNA-seq (Fig. S4, *B* and *C*). These data suggest that the translatome undergoes more extensive remodeling than the transcriptome during totipotent-like reprogramming.

We next examined the quantitative dynamics of global translation. Although some transcripts showed statistically significant changes, the overall transcriptome varied within a relatively narrow range (median log_2_Transcripts Per Kilobase Million (TPM) between 2.37 and 2.58), whereas both ribosome density and translational efficiency (TE) were markedly suppressed at 12 h, followed by a partial recovery by 24 h (Fig. 1*E*). To functionally validate this reprogramming-associated translational repression, we directly measured nascent protein synthesis using the SUnSET assay. Consistent with the above observations, puromycin signal was strongly reduced at 12 h and modestly recovered at 24 h, whereas control cells lacking the Dox-inducible *Dux* system showed no notable change upon Dox treatment, confirming that the observed repression was not attributable to Dox itself (Fig. 1*F*). Together, these results reveal translational activity as a dynamically regulated layer during totipotent-like reprogramming.

### The reprogramming signal couples mTORC1 inhibition to suppressed translation initiation

**Figure 2:**
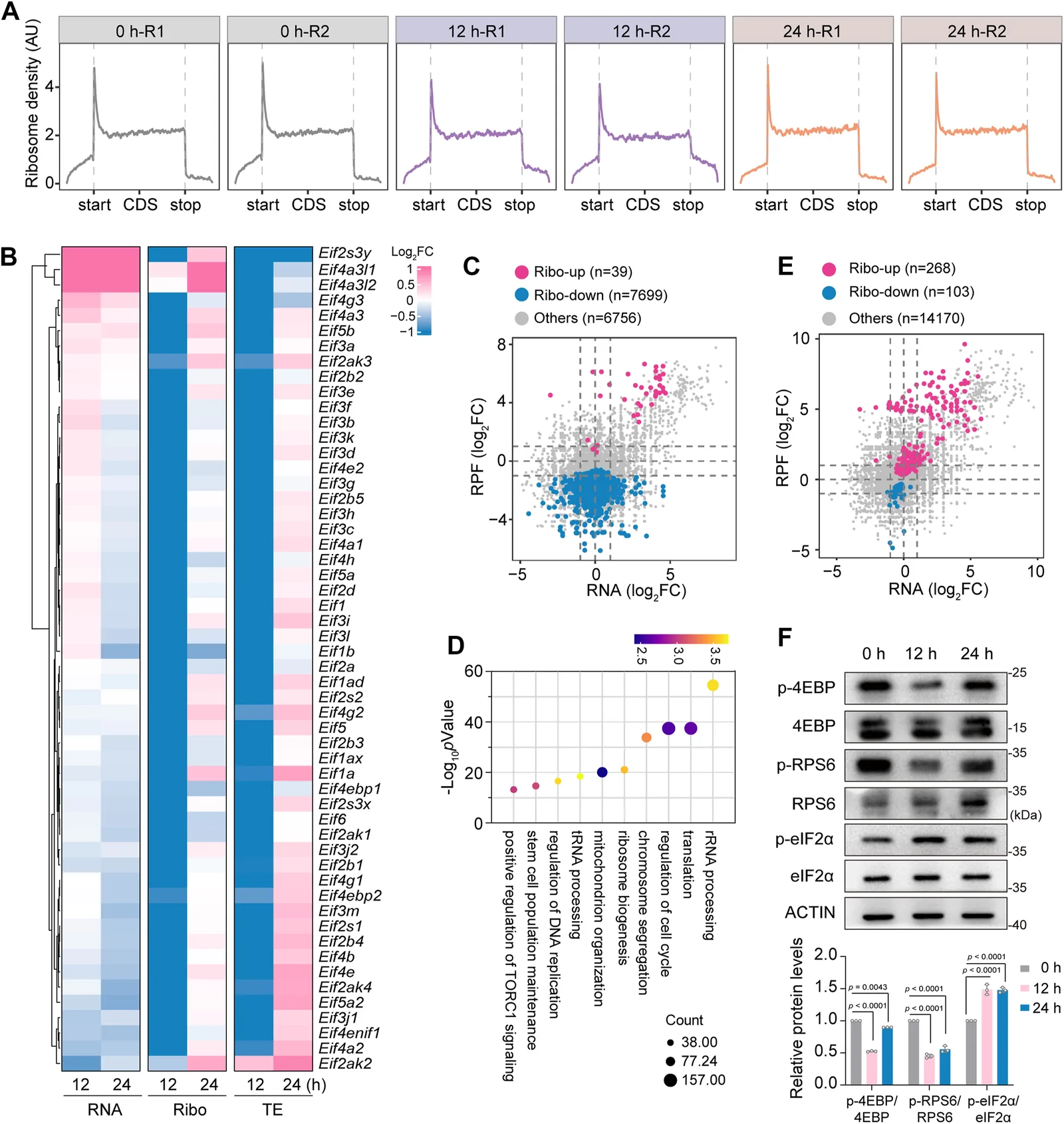
**Reprogramming signal deactivates mTORC1 signaling and inhibits translation initiation.***A,* metagene analysis of average ribosome density across mRNA regions of all annotated transcripts during reprogramming. *B,* heatmap showing the log_2_FC of mRNA abundances, ribosome densities, and translation efficiencies for initiation factors at 12 h and 24 h relative to 0 h. The color scale indicates the magnitude of *log*_*2*_*FC (red, upregulation; blue, downregulation)*. *C,* scatter plots comparing gene-level changes in mRNA abundance (RNA log_2_FC) *versus* ribosome density (RPF log_2_FC) at 12 h post-induction relative to 0 h. Each dot represents an individual gene. Genes with significant changes in RPF (|log_2_FC| > 0, *p* < 0.05) but no significant change in RNA are highlighted in *red* (Ribo-up) or *blue* (Ribo-down), whereas all others are shown in *gray*. This classification identifies genes whose expression changes are primarily driven by translational regulation without transcriptional confounding. The complete gene lists for each category are provided in Table S2. *D,* Gene Ontology enrichment analysis for biological processes associated with genes exhibiting significantly decreased TE (Ribo-down) at 12 h. *E,* scatter plots comparing gene-level changes in mRNA abundance *versus* ribosome density at 24 h post-induction relative to 0 h. *F,* immunoblot analysis of mTOR pathway-related and ISR pathway-related regulators during totipotent-like reprogramming. *Top*: representative blots showing the phosphorylation and total protein levels of 4EBP1, RPS6, and eIF2α. *Bottom*: quantification of p-4EBP/4EBP, p-RPS6/RPS6, and p-eIF2α/eIF2α ratios, normalized to the 0 h control. Data are presented as mean ± S.D. from independent biological replicates (n = 3), exact *p* values were included, one-way ANOVA with Dunnett's multiple comparisons test. log2FC, log2fold change; TE, translational efficiency.

Having established the occurrence of global translational suppression during early totipotent-like reprogramming, we next aimed to delineate the specific alterations in the translational machinery underlying this phenomenon. To identify which specific stages of translation were altered, we first analyzed the genome-wide distribution of RPFs across mRNA regions. Metagene analysis revealed a profound depletion of RPFs at the translation initiation site at 12 h post-induction, which partially recovered by 24 h. In contrast, ribosome occupancy across the coding sequence and near the stop codon remained largely unaltered (Figs. 2*A* and S5*A*). This specific defect in initiation provides a mechanistic basis for the global translational reduction.

Given the implication of impaired translation initiation, we next systematically examined the expression dynamics of translation initiation factors. Intriguingly, while the mRNA levels of most translation initiation factors remained largely stable, ribosome profiling revealed a marked decrease in their translation at 12 h, followed by substantial recovery at 24 h (Fig. 2*B*). Notably, this suppression extended coherently to genes encoding elongation and termination factors, all exhibiting coordinated translational downregulation at 12 h (Fig. S5, *B* and *C*). These results demonstrate that early reprogramming triggers a broad, translation-level repression of the entire protein synthesis apparatus.

To comprehensively evaluate the extent and specificity of translational regulation, we performed an integrated comparison of RNA-seq and Ribo-seq data by plotting the fold-change in ribosome occupancy (RPFs) against the corresponding mRNA fold-change for each gene. This analysis identified 7699 genes exhibiting translational repression independent of transcriptional change at 12 h (Fig. 2*C*). Gene Ontology (GO) enrichment analysis revealed that these genes were highly enriched in translation and cell cycle regulation (Fig. 2*D*), indicating targeted downregulation of core protein synthesis and proliferative functions. This suppression was largely transient, with ribosome occupancy restored by 24 h. In contrast, only 39 genes exhibited significant translational upregulation at 12 h, a number that increased to 268 by 24 h (Fig. 2*E*), suggesting a delayed but selective activation of specific programs.

The profound but transient suppression of global protein synthesis suggests that cells enter a state of translational arrest during early stage of totipotent-like reprogramming. To identify upstream signals, we examined key translation-related pathways. GO analysis of translationally suppressed genes at 12 h also revealed significant enrichment for mTORC1 signaling (Fig. 2*D*). Consistently, immunoblotting showed decreased phosphorylation of the mTORC1 targets 4E-BP1 and RPS6 at 12 h, indicating suppressed cap-dependent translation initiation. By 24 h, phosphorylation levels of these proteins largely recovered. Concurrently, eIF2α Ser51 phosphorylation, a well-established regulatory node of translation initiation, was significantly elevated (Fig. 2*F*). These phosphorylation changes were specific to the reprogramming process, as they were absent in control cells lacking the Dox-*Dux* system upon Dox treatment (Fig. S5*D*). Together, these findings indicate that early reprogramming activates two convergent pathways, mTORC1 suppression and eIF2α phosphorylation, to enforce a global translational arrest, potentially suspending existing cellular programs to facilitate proteome remodeling.

### Selective upregulation of mitochondrial translation is required for totipotent-like reprogramming

**Figure 3:**
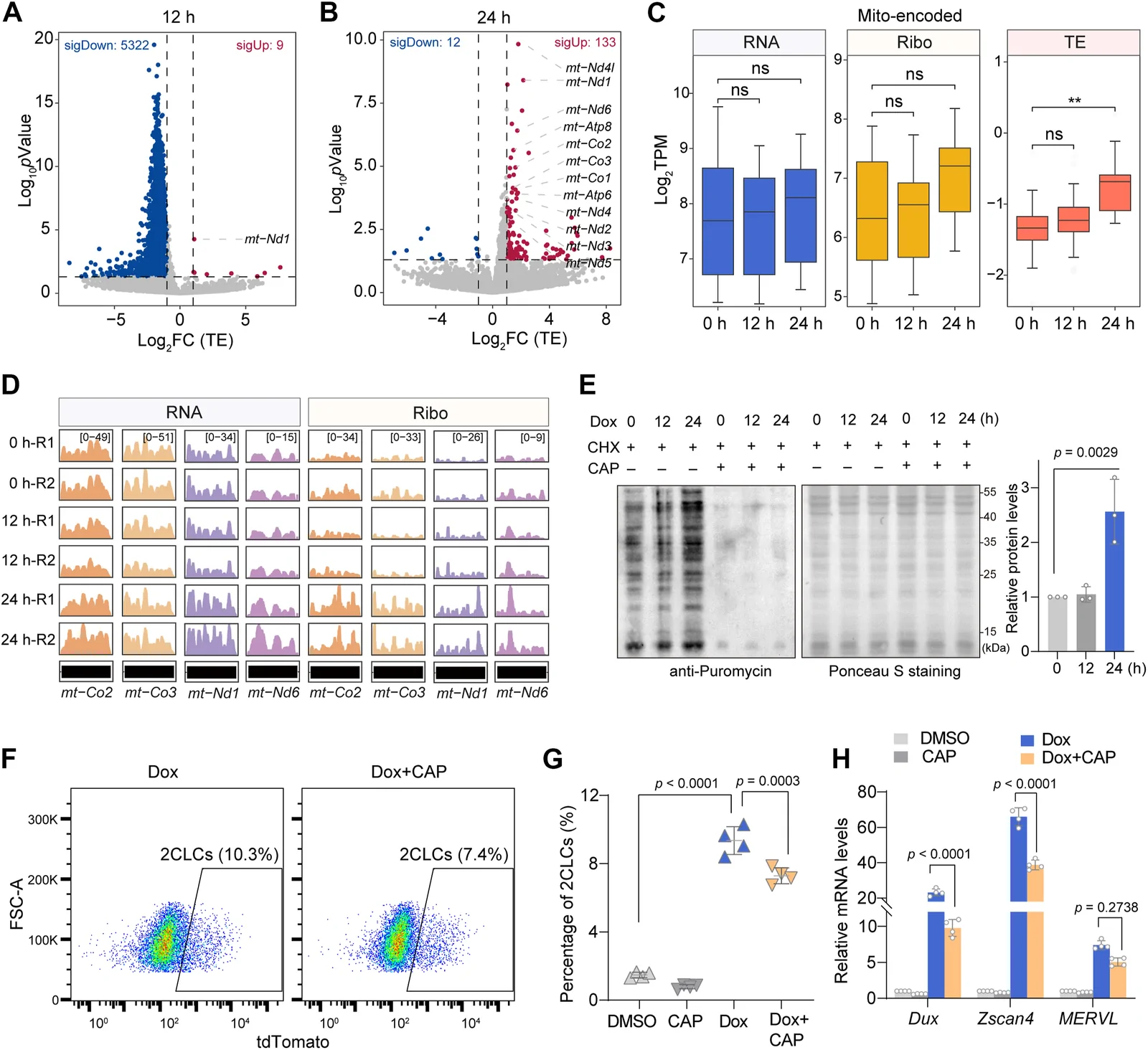
**Mitochondrial translation is selectively upregulated to promote totipotent-like reprogramming.***A and B, volcano plots* showing differentially expressed genes in TE at 12 h *(A)* and 24 h *(B)* post-induction relative to the 0-h control. Upregulated genes (fold change > 2, *p* < 0.05) are marked in *red,* while downregulated genes (fold change < 0.5, *p* < 0.05) are marked in *blue*. *C, Boxplots* comparing the expression levels of the 13 mitochondrial genome-encoded genes across different timepoints, as measured by mRNA abundance, ribosome density, and calculated TE. Statistical significance was determined by Wilcoxon test (∗∗*p* < 0.01). *D,* genome browser view of representative regions for representative mitochondrially encoded genes (*mt-Co2*, *mt-Co3*, *mt-Nd1*, *mt-Nd6*) in cells across the three timepoints during reprogramming. *E*, mitochondrial protein synthesis in ESCs was measured by SUnSET assay during totipotent-like reprogramming. *Left*: Representative blots showing puromycin incorporation at 0 h, 12 h, and 24 h under CHX pretreatment (lanes 1, 2, 3) and CHX + CAP co-treatment (lanes 4, 5, 6). Ponceau S staining serves as a loading control. *Right*: Quantification of puromycin incorporation signals, normalized to the 0 h control. Data are presented as mean ± S.D. from independent biological replicates (n = 3), exact *p* values were included, one-way ANOVA with Dunnett's multiple comparisons test. *F,* flow cytometry analysis showing the percentage of 2C-tdTomato-positive cells in the absence or presence of CAP (100 μg/ml, 24 h). *G,* quantification of the percentage of 2C-tdTomato-positive cells in the absence or presence of CAP from four biologically independent experiments. *H,* detection of 2C gene expression by RT-qPCR in the absence or presence of CAP. Data are presented as mean ± S.D. from independent biological replicates (n = 4); exact *p* values were included, one-way ANOVA with Tukey's multiple comparisons test. CHX, cycloheximide; TE, translational efficiency.

Since global translation is broadly suppressed during the early phase of reprogramming, we hypothesized that genes escaping such translational inhibition or being selectively upregulated at this level might play pivotal roles in directing cell fate transition. To systematically identify these genes, we performed a transcriptome-wide differential analysis of TE. Volcano plot revealed a striking temporal asymmetry: only nine genes exhibited significantly increased TE at 12 h, whereas 133 genes showed significant TE upregulation at 24 h (Fig. 3, *A* and *B*). GO analysis of these 24 h targets highlighted a strong enrichment for mitochondrial functions, most notably oxidative phosphorylation (Fig. S6*A*).

To quantitatively assess this selective activation, we analyzed the expression dynamics of the 13 mitochondrially encoded genes. Although their mRNA levels remained mildly changed, both ribosome density and TE were obviously elevated at 24 h (Fig. 3*C*), demonstrating that mitochondrial translation is specifically upregulated in the context of global cytosolic suppression. Representative mitochondrially encoded genes, including *mt-CO2*, *mt-CO3*, and *mt-ND6*, showed pronounced TE elevation by 24 h, with *mt-ND1* already exhibiting selective translational upregulation as early as 12 h (Fig. 3*D*). Consistent with this, these 13 mitochondrially encoded genes exhibited markedly higher TE at the 2-cell and 4-cell stages than at the blastocyst stage in mouse early embryos (Fig. S6*B*). These results indicate that mitochondrial translation is selectively and progressively activated during totipotent-like reprogramming, a feature also observed in early embryonic development.

To obtain direct biochemical validation, we employed nascent protein labeling coupled with selective translational inhibitors to monitor *de novo* mitochondrial protein synthesis, adapting an approach from a previous report (30). Cells were pretreated with cycloheximide (CHX) to inhibit cytosolic translation, ensuring that subsequent puromycin labeling primarily reflected mitochondrial translation activity. The specificity of this signal was confirmed by its abolition upon cotreatment with the mitochondrial translation inhibitor chloramphenicol (CAP). Using this approach, we observed that *de novo* mitochondrial protein synthesis was low at 0 and 12 h but increased markedly at 24 h (Fig. 3*E*), consistent with the Ribo-seq data. Together, these results demonstrate that mitochondrial translation is selectively upregulated during reprogramming. To functionally assess the importance of this selective upregulation, we inhibited mitochondrial protein synthesis using CAP during totipotent-like reprogramming. Flow cytometry analysis revealed that CAP treatment led to a significant reduction in the percentage of tdTomato^+^ cells (Fig. 3, *F* and *G*). Correspondingly, the expression of key 2C markers (*Dux*, *Zscan4*, *MERVL*) was markedly diminished upon mitochondrial translation inhibition (Fig. 3*H*). To distinguish whether this effect reflects a specific requirement for mitochondrial translation in the reprogramming process rather than general mitochondrial dysfunction that impairs stem cell maintenance, we repeated CAP treatment under 2i/leukemia inhibitory factor (LIF) conditions, which stabilize ESCs in the naïve pluripotent state. Notably, CAP similarly reduced Dox-induced 2CLC formation and suppressed the upregulation of 2C markers, without increasing background 2CLC levels (Fig. S6, *C*–*E*). These results demonstrate that elevated mitochondrial translation is functionally essential for the acquisition of the totipotent-like state.

### Mitochondrial remodeling during reprogramming features the content and activity reduction

**Figure 4:**
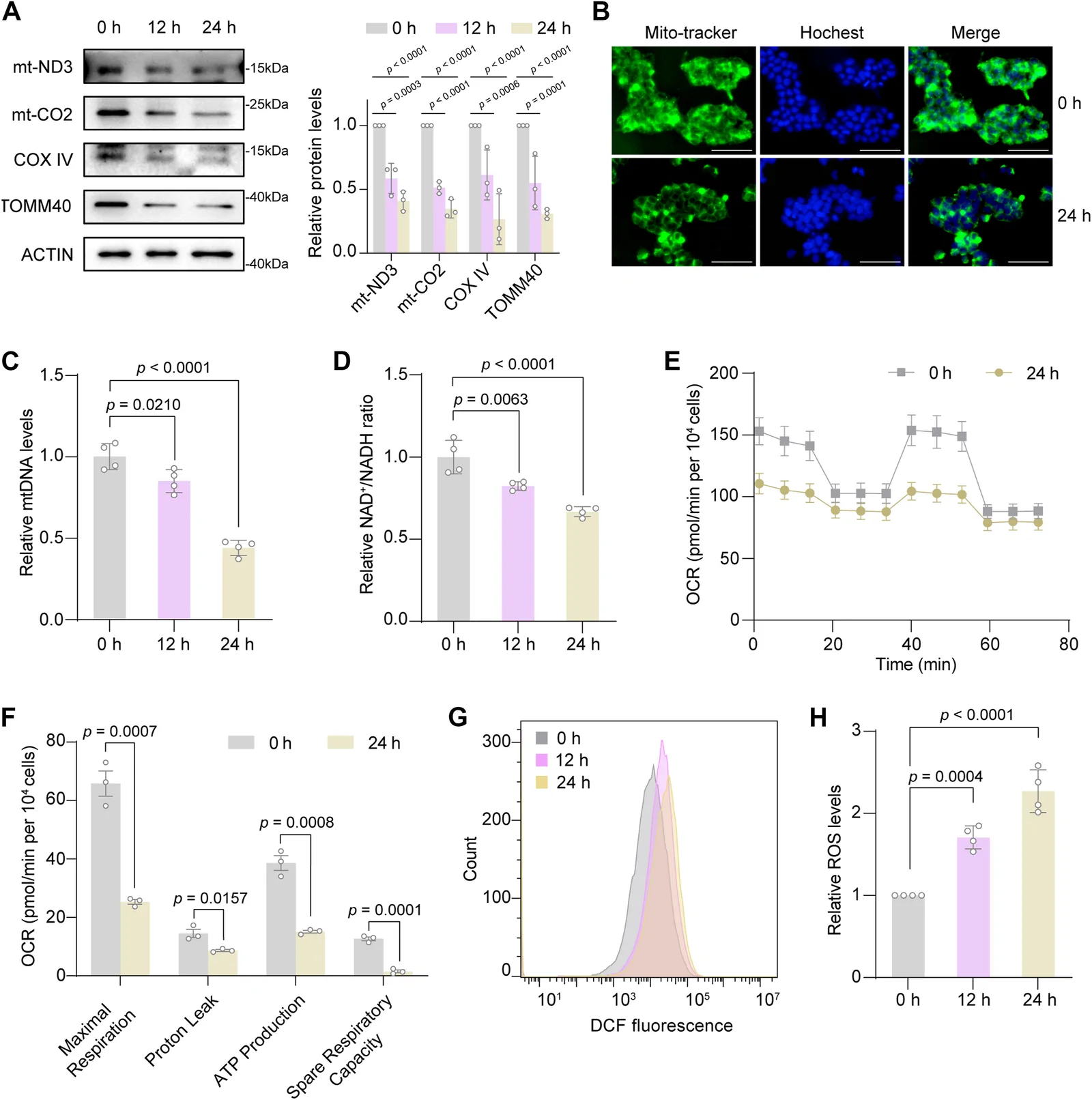
**Reprogramming drives the reduction of mitochondrial content and activity.***A,* immunoblot analysis of mitochondrial protein abundance during reprogramming. *Left*: representative blots showing the protein levels of mitochondria-encoded proteins (mt-CO2, mt-ND3) and nuclear-encoded mitochondrial proteins (COX IV, TOMM40). ACTIN is shown as a loading control. *Right*: quantification of the protein levels, normalized to the 0 h control. Data are presented as mean ± S.D. from independent biological replicates (n = 3), exact *p* values were included, one-way ANOVA with Dunnett's multiple comparisons test. *B,* representative fluorescence microscopy images of cells stained with Mito-Tracker *green* at 0 and 24 h postinduction. The scale bar represents 5 μm. *C,* detection of mitochondrial DNA copy number by qPCR in ESCs during reprogramming. Data are mean ± S.D.; exact *p* values were included, one-way ANOVA with Dunnett's multiple comparisons test; n = 4. *D*, measurement of the cellular NAD^+^/NADH ratio in ESCs at different timepoints. Data are mean ± S.D.; exact *p* values were included, one-way ANOVA with Dunnett's multiple comparisons test; n = 4. *E,* measurement of the mitochondrial oxygen consumption rate in ESCs at 0 and 24 h (n = 3). *F*, quantification of oxygen consumption rate parameters, including maximal respiration, proton leak, ATP production, and spare respiratory capacity. Data are mean ± S.D.; exact *p* values were included, two-tailed Student’s *t* test; n = 3. *G,* flow cytometry histograms showing the fluorescence intensity of cells stained with the ROS-sensitive probe DCFH-DA at 0, 12, and 24 h. *H,* quantification of the relative ROS levels in ESCs. Data are mean ± S.D.; exact *p* values were included, one-way ANOVA with Dunnett's multiple comparisons test; n = 4. ESC, embryonic stem cell; ROS, reactive oxygen species.

Given the observed upregulation of mitochondrial TE, we next examined mitochondrial protein abundance during totipotent-like reprogramming. Surprisingly, immunoblot analysis revealed that total cellular levels of mitochondrially-encoded proteins (*e.g.*, mt-ND3 and mt-CO2) progressively decreased throughout the process. This reduction extended to nuclear-encoded mitochondrial proteins, including COX IV and TOMM40 (Fig. 4*A*). These changes were absent in control cells lacking the Dox-*Dux* system upon Dox treatment (Fig. S6*F*), confirming their specificity to the reprogramming process. Thus, despite enhanced translation of mitochondrially-encoded subunits, mitochondrial proteins were broadly downregulated, prompting us to hypothesize that overall mitochondrial content is systematically reduced during the transition toward a totipotent-like state.

To directly test this hypothesis, we employed two independent approaches. First, MitoTracker fluorescence staining showed a pronounced decrease in mitochondrial signal intensity by 24 h compared to the 0-h control, indicating a reduction of mitochondrial mass (Fig. 4*B*). Second, quantitative PCR analysis revealed a progressive, time-dependent decline in mitochondrial DNA (mtDNA) copy number per cell over the reprogramming time course (Fig. 4*C*). Together, the reduction in both MitoTracker fluorescence and mtDNA copy number provides evidence that mitochondrial content is systematically diminished during totipotent-like reprogramming.

We then investigated whether the observed reduction in mitochondrial content correlates with altered functional capacity. To evaluate mitochondrial metabolic activity, we first measured the cellular NAD^+^/NADH ratio, a key indicator of the oxidative phosphorylation (OXPHOS) redox state. This ratio progressively declined during reprogramming (Fig. 4*D*). We next directly quantified mitochondrial respiratory function using Seahorse assay-based measurements of the oxygen consumption rate (OCR). Consistent with the NAD^+^/NADH data, basal OCR, as well as maximal and ATP-linked respiration, was significantly lower at 24 h compared to the 0-h baseline (Fig. 4, *E* and *F*). These results demonstrate a pronounced downregulation of OXPHOS capacity. Concurrently, intracellular reactive oxygen species (ROS) levels showed a time-dependent increase (Fig. 4, *G* and *H*), consistent with impaired electron transport chain function. Taken together, our findings reveal that reprogramming of ESCs to 2CLCs is accompanied by extensive mitochondrial remodeling, with 2CLCs exhibiting enhanced mitochondrial translation despite a net reduction in mitochondrial content and OXPHOS capacity compared to ESCs.

## Discussion

**Figure 5:**
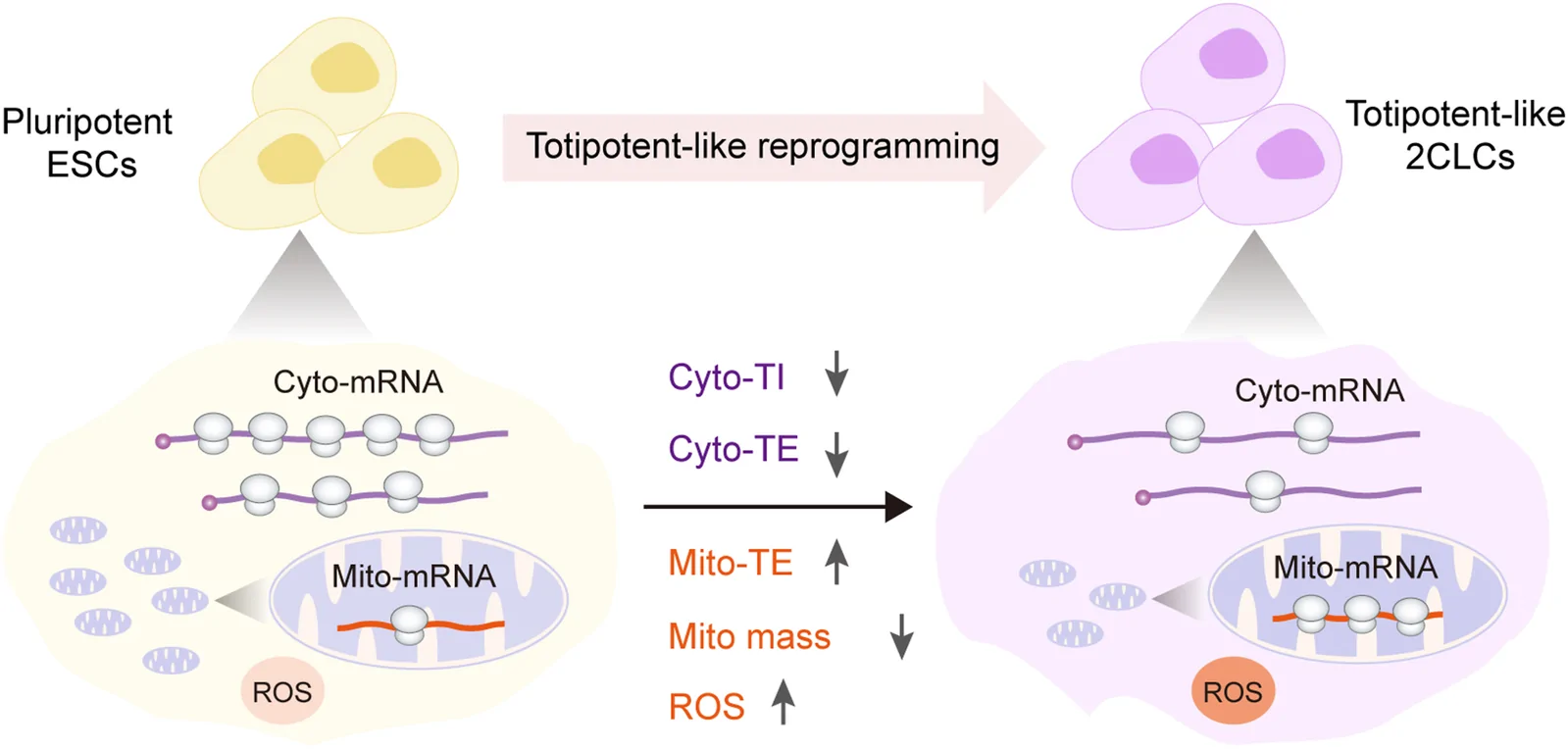
**Translational dynamics during totipotent-like reprogramming.** Working model showing the dynamic translatome dynamics featuring decreased cytosolic translation and upregulated mitochondrial translation, which is associated with mitochondrial remodeling during totipotent-like reprogramming.

2CLCs constitute an invaluable totipotent-like cellular model for elucidating the molecular mechanisms governing totipotency establishment, as they recapitulate key molecular and metabolic characteristics of the totipotent 2-cell blastomere and retain the capacity to differentiate into both embryonic and extraembryonic lineages (4, 31). To date, research on totipotent-like reprogramming has predominantly focused on transcriptional regulators, RNA modulators, and epigenetic modifications, whereas the role of translational regulation in this process remains largely unexplored. Here, we present the first comprehensive translatome analysis during the reprogramming of pluripotent ESCs to totipotent-like 2CLCs using Ribo-seq. Our results demonstrate a profound yet transient suppression of global protein synthesis, followed by the selective translational upregulation of a specific subset of genes (Fig. 5). This translational reprogramming defines a critical and previously uncharacterized regulatory layer essential for the acquisition of a totipotent-like state in mouse ESCs.

Translation initiation serves as the rate-limiting and stringently regulated stage of gene expression (32). eIF2α and mTORC1 function as central regulators of mRNA translation by modulating this initiation step, enabling cells to respond to diverse intracellular and environmental stresses (27, 33, 34). Analysis of ribosome distribution revealed a specific decrease in ribosome density at translation start sites during early reprogramming, indicating that the observed global suppression of protein synthesis primarily originates from inhibition at the translation initiation level (Fig. 2*A*). We further identified that the reprogramming signal triggers two major events, phosphorylation of eIF2α and inhibition of the mTORC1 pathway (Fig. 2*F*). These likely operate as a coordinated dual-brake system that potently suppresses translation initiation. The partial recovery of translation by 24 h suggests this mechanism constitutes a regulated, adaptive pause rather than a terminal shutdown. Based on these dynamic translational features, we propose that this programmed translational pause may facilitate a temporary suspension of the pluripotent proteome, allowing for subsequent production of proteins essential for establishing the totipotent-like state.

Despite global translational suppression, we observed a selective upregulation of mitochondrial translation (Fig. 3, *A*–*E*). Segregated from cytosolic translation both physically and genetically, mitochondria retain a semi-autonomous system dedicated to synthesizing the 13 polypeptides encoded by their genome. The two translation systems exhibit distinct regulatory paradigms in response to cellular signals. Cytosolic translation is highly dynamic and rapidly responsive, allowing cells to adjust the proteome within minutes. In contrast, mitochondrial translation is regulated through slower, multi-layered mechanisms (35). We propose that this architectural and regulatory independence may allow mitochondrial translation to remain partially insulated from the acute repressive signals that globally inhibit cytosolic initiation during early totipotent-like reprogramming. Notably, the selective upregulation of mitochondrial translation observed in our 2CLC model is consistent with the physiological mitochondrial dynamics during early mouse embryonic development (Fig. S6*B*). This concordance supports the physiological relevance of our findings and suggests that enhanced mitochondrial translation may be a conserved feature associated with the totipotent state.

The selective upregulation of mitochondrial translation, however, coincides with an overall reduction in mitochondrial content, indicating that active organellar remodeling accompanies this profound cell identity transition. This is consistent with the physiological dynamics of mitochondrial respiratory activity during early mouse embryonic development, where oxygen consumption is relatively low at the cleavage stage and increases significantly towards the blastocyst stage. Remodeling of mitochondrial mass and activity has been observed during cell fate transition. For instance, during white-to-beige adipocyte differentiation, cells undergo extensive mitochondrial biogenesis and proteome rewiring to meet new metabolic demands (36). In contrast, totipotent-like reprogramming initiates a molecularly distinct remodeling program. A critical output of this imbalanced remodeling is the generation of ROS. Notably, a recent study indicates that heterogeneity in mitochondrial ROS levels among mouse ESCs dictates lineage choice, with higher ROS predisposing cells to mesendoderm differentiation (37). Furthermore, controlled ROS signaling is a well-recognized mediator in stem cell maintenance and reprogramming (38). Therefore, the ROS increase observed here likely functions as a physiological signaling molecule that facilitates the cell state transition, rather than a mere byproduct of metabolic damage.

In summary, our study reveals that coordinated translational control and mitochondrial reprogramming are crucial for establishing cellular totipotency, providing a novel dimension to our understanding of cell fate determination.

## Experimental procedures

### Cell culture

ESCs were cultured on plates coated with 0.2% gelatin in KnockOut DMEM (Gibco) supplemented with 15% KnockOut Serum Replacement (Gibco), 1% non-essential amino acids (Gibco), 1% GlutaMAX (Gibco), 1% penicillin-streptomycin (Life Technologies), 0.1 mM β-mercaptoethanol (Invitrogen), and 1000 U/ml recombinant mouse LIF (Millipore). Cultures were maintained at 37 °C in a humidified atmosphere of 5% CO_2_. The medium was changed daily, and cells were routinely passaged when they reached approximately 70% confluency. For 2i/LIF conditions, ESCs were cultured in the same basal medium additionally supplemented with 3 μM CHIR99021 (Laduviglusib, MedChemExpress) and 1 μM PD0325901 (Mirdametinib, MedChemExpress) to maintain the naïve pluripotent state. Cells were adapted to 2i/LIF for at least two passages prior to experimental treatments. All cell lines used in this study were tested for *mycoplasma* contamination and confirmed to be negative prior to experiments.

### Induction of totipotent-like reprogramming

For reprogramming induction, ESCs were passaged and seeded at a density of approximately 50% confluency, as appropriate for downstream applications. After 12 h of attachment, cells were treated with Dox(Sigma) at a final concentration of 2 μg/ml to induce expression of the *Dux* transgene. Cells were harvested at the indicated time points (0 h, 12 h, and 24 h) for subsequent analyses. For control groups, cells were treated with an equivalent volume of DMSO (vehicle control).

### Flow cytometry analysis

Twenty-four hours prior to analysis, ESCs were seeded into 35-mm culture dishes. For sample preparation, cells were dissociated using 0.05% trypsin-EDTA (Gibco), centrifuged to remove the culture medium, washed once with 1 ml of 1 × PBS, and resuspended in PBS. The cell suspension was then passed through a 300-mesh sieve into a flow cytometry tube and analyzed immediately on a FACSCelesta flow cytometer (Becton Dickinson). Data analysis was performed using FlowJo software (version 10.8; https://www.flowjo.com).

### RNA isolation and real-time quantitative PCR

Total RNA was extracted from cells using TRIzol reagent (Invitrogen) following the manufacturer’s protocol. Potential genomic DNA contamination was removed using the gDNA wiper mix, and reverse transcription was performed with HiScript III RT SuperMix for qPCR (+gDNA wiper) (Vazyme). Quantitative real-time PCR (qPCR) was carried out using ChamQ SYBR qPCR Master Mix (Low ROX Premixed; Vazyme Biotech) on a QuantStudio 3 Real-Time PCR System (Applied Biosystems). Gene expression levels were normalized to β-actin as the internal reference, and relative quantification was calculated using the 2^−ΔΔCt^ method. All primer sequences used are provided in Table S1.

### SUnSET assay

Cells were seeded in 35-mm dishes at a density of 2 × 10^5^ cells per dish and allowed to grow until 70 to 80% confluency was reached. After incubation in fresh medium for 2 h, cells were treated with 10 μg/ml puromycin for 15 min to label newly synthesized polypeptides. Following two washes with ice-cold PBS, cells were lysed directly in SDS-PAGE loading buffer. Puromycin incorporation was detected by Western blot using an anti-puromycin antibody (Sigma-Aldrich, MABE343).

For mitochondrial nascent protein synthesis, cells were pretreated with 100 μg/ml CHX (MedChemExpress) for 2 h to inhibit cytosolic translation, followed by puromycin labeling (10 μg/ml, 15 min) to specifically label mitochondrial translation products. As a specificity control, cells were co-treated with 100 μg/ml chloramphenicol (CAP, MedChemExpress) together with CHX prior to puromycin labeling. Cells were then lysed and subjected to Western blot analysis using the anti-puromycin antibody.

### Western blot

Total proteins were extracted by lysing cells in 2 × SDS loading buffer, followed by denaturation at 100 °C for 10 min. Equal amounts of protein lysates were separated on 8 to 12% SDS-polyacrylamide gels and transferred onto nitrocellulose membranes (Sangon Biotech). The membranes were blocked with 5% non-fat milk in TBST for 1 h at room temperature and then incubated overnight at 4 °C with primary antibodies. After three washes with TBST, membranes were incubated for 1 h at room temperature with corresponding horseradish peroxidase-conjugated secondary antibodies. Signals were detected using enhanced chemiluminescence substrate (Vazyme, E412-02) and imaged with a Tanon imaging system (Tanon Science & Technology). Antibodies used in the experiments were presented as below: anti-4EBP1 (Abclonal, A1248), anti-p-4EBP1 (Abclonal, AP0030), anti-RPS6 (Abclonal, A11874), anti-p-RPS6 (Abclonal, AP1325), anti-eIF2α (Santa Cruz, sc-133132), anti-p-eIF2α (CST, 3398T), anti-MT-ND3 (Abclonal, A9940), anti-MTCO2 (Abclonal, A11522), anti-COXIV (Proteintech, 11242-1-AP), anti-TOMM40 (Proteintech, 18409-1-AP). Band intensities were quantified using Tanon Image Analysis Software (https://www.biotanon.com) and normalized to the corresponding loading control to determine relative protein levels.

### MitoTracker green fluorescence assay

Cells were seeded onto gelatin-coated six-well plates and allowed to adhere. Mitochondrial mass was assessed using Mito-Tracker Green (Beyotime, C1048). Following two washes with prewarmed (37 °C) PBS, cells were incubated with 500 nM Mito-Tracker Green in KnockOut DMEM (Gibco) for 30 min at 37 °C. After removal of the staining solution, cells were washed twice with PBS. Nuclei were counterstained with Hoechst 33258 (Santa Cruz). Fluorescence images were captured using an inverted fluorescence microscope (Nikon ECLIPSE Ts2).

### Mitochondrial DNA quantification

Total cellular DNA was extracted using the FastPure Cell DNA Isolation Mini Kit (Vazyme, DC102-01) according to the manufacturer’s instructions. mtDNA content was quantified by real-time PCR with the Mouse mtDNA Copy Number qPCR Kit (Beyotime, D8033S). Reactions were performed in a total volume of 10 μl containing template DNA and the provided primer mix. The thermal cycling protocol consisted of initial steps at 50 °C for 5 min and 95 °C for 2 min, followed by 40 cycles of denaturation at 95 °C for 15 s and annealing/extension at 55 °C for 15 s. Relative mtDNA copy number was normalized to a nuclear-encoded reference gene using the 2^−ΔΔCt^ method.

### NAD^+^/NADH measurements

Cellular NAD^+^ and NADH levels were determined using a commercial NAD^+^/NADH Assay Kit with WST-8 (Beyotime, S0175) according to the manufacturer's protocol. Briefly, cells were seeded in 35-mm dishes at a density of 2 × 10^5^ cells per dish and cultured overnight. After two washes with PBS, cells were lysed with 200 μl of ice-cold extraction solution provided in the kit. The lysate was centrifuged at 12,000×*g* for 10 min at 4 °C, and the supernatant was collected for subsequent assays. For the measurement of total NAD^+^, 20 μl of supernatant was transferred to a 96-well plate and mixed with 90 μl of alcohol dehydrogenase working solution. To specifically quantify NADH, a separate 50 μl aliquot of lysate was heated at 60 °C for 30 min to decompose NAD^+^, after which 20 μl of the heat-treated sample was used for the assay. Absorbance was measured using a microplate reader (BioTek, SYNERGY H1). NAD^+^ and NADH concentrations were calculated based on standard curves and normalized to the total protein content of each sample.

### Oxygen consumption rate measurements

Mitochondrial respiratory function was assessed using the Seahorse XF96 Cell Mito Stress Test Kit (Agilent) on an XF96 Extracellular Flux Analyzer (Agilent) following the manufacturer's instructions. Briefly, cells were seeded into XF96 cell culture microplates at a density of 5,000 cells per well and allowed to adhere overnight. On the day of the assay, the culture medium was replaced with Seahorse XF DMEM assay medium (pH 7.4) supplemented with 10 mM glucose, 1 mM sodium pyruvate, and 2 mM L-glutamine. The sensor cartridge was hydrated in XF calibrant solution at 37 °C in a non-CO_2_ incubator overnight and equilibrated for at least 1 h prior to the assay. Mitochondrial respiration was measured by sequentially injecting the following inhibitors: 1.5 μM oligomycin (ATP synthase inhibitor), 1.5 μM carbonyl cyanide-4-(trifluoromethoxy) phenylhydrazone (FCCP; uncoupler), and a mixture of 0.5 μM rotenone and 0.5 μM antimycin A (complex I and III inhibitors). OCR was recorded in real time. Data were acquired using Wave software (Agilent Technologies; https://www.agilent.com) and analyzed for basal respiration, ATP-linked respiration, maximal respiration, and spare respiratory capacity.

### Intracellular ROS production assays

Intracellular ROS levels were measured using a fluorescent probe-based ROS Assay Kit (Beyotime, S0033S) in accordance with the manufacturer's instructions. Briefly, cells were seeded in 35-mm dishes at a density of 2 × 10^5^ cells per dish and cultured overnight. Prior to the assay, cells were washed once with prewarmed PBS and then incubated with KnockOut DMEM (Gibco) containing 10 μM DCFH-DA for 30 min at 37 °C in the dark. Following incubation, cells were washed twice with PBS, detached using trypsin-EDTA, and resuspended in PBS for immediate analysis. Fluorescence intensity corresponding to intracellular ROS was quantified using a flow cytometer (BD, FACSCelesta). Data from at least 10,000 events per sample were collected and analyzed using FlowJo software (version 10.8).

### Ribo-seq and input RNA-seq

For ribosome profiling and matched input RNA sequencing, cells were grown to 70 to 80% confluence. Prior to harvest, the medium was replaced with fresh medium for 2 h, followed by a 7 min treatment with 100 μg/ml CHX to arrest translating ribosomes. Cells were then lysed on ice for 20 min using polysome lysis buffer (10 mM HEPES pH 7.4, 5 mM MgCl_2_, 100 mM KCl, 1% Triton X-100, 1 mM DTT, 200 μg/ml CHX). Lysates were cleared by centrifugation at 15,000 rpm for 10 min at 4 °C. One-sixth of the supernatant was reserved as the input control for total RNA-seq. RNA was extracted from this aliquot using TRIzol LS (Invitrogen) and subsequently fragmented in freshly prepared fragmentation buffer (100 mM Tris-HCl pH 7.0, 100 mM ZnCl_2_) at 94 °C for 4 min. For ribosome-protected fragment (RPF) isolation, the remaining supernatant was digested with 3 μl of RNase I (Thermo Fisher Scientific, AM2294) for 1 h at 4 °C with gentle rotation. The digested lysate was then applied to MicroSpin S-400 HR columns (GE Healthcare, 27-5140-01) for size-exclusion chromatography according to the manufacturer's instructions. The flow-through containing ribosome-protected RNA fragments was collected by centrifugation. RNA was then extracted from the flow-through using three volumes of TRIzol LS.

For cDNA library construction and high-throughput sequencing, both input (fragmented) and RPF RNA samples were size-selected on a 15% denaturing polyacrylamide TBE-urea gel. RNA fragments of 28 to 32 nt (for Ribo-seq) and 40 to 60 nt (for input RNA-seq) were excised, eluted overnight in gel extraction buffer (300 mM NaOAc pH 5.5, 1 mM EDTA, 0.1 U/μl SUPERase-In), and ethanol-precipitated. Purified RNA was dephosphorylated and then ligated to a preadenylated 3′ adapter using T4 RNA Ligase 1 (NEB, M0437M). Following reverse transcription with Superscript III (Invitrogen), the cDNA was circularized and PCR-amplified for 15 cycles with Phusion High-Fidelity DNA Polymerase (NEB, M0530L) using indexed primers. DNA was extracted and dissolved in 21 μl of enzyme-free water, of which 1 μl was evaluated on the Agilent Bioanalyzer 2100 system, and sent for sequencing on an Illumina NovaSeq platform at Annoroad.

### Ribo-seq data processing

For paired-end Ribo-seq sequencing, R1 reads were utilized for all analyses. Raw Ribo-seq reads were initially processed using Cutadapt (v2.8; https://cutadapt.readthedocs.io) to remove adapter sequences with the following parameters: --match-read-wildcards -a NNNNNNNNNNAGATC GGAAGAGCACACGTC -m 20 -M 35 --discard-untrimmed. The same procedure was applied to RNA-seq reads, but without the maximum length constraint, retaining reads with a minimum length of 20 (-m 20). Quality-filtered reads were sequentially aligned to mouse rRNA and tRNA reference sequences using Bowtie2 (v2.3.5.1; https://github.com/BenLangmead/bowtie2) to eliminate contaminating reads. The remaining non-rRNA/non-tRNA reads were then aligned to the mouse reference genome (GRCm38) using HISAT2 (v2.2.1; https://daehwankimlab.github.io/hisat2) with -k 5 parameter. Resulting alignments were converted to BAM format and sorted using SAMtools (v1.21; https://www.htslib.org). The 5′ ends of the Ribo-seq reads were used for downstream analysis. Read counts were quantified using featureCounts (v2.0.6; https://subread.sourceforge.net), with features specified as exons (-t exon) for RNA-seq and coding sequences (-t CDS) for Ribo-seq. TE was calculated as the ratio of TPM-normalized Ribo-seq to TPM-normalized RNA-seq data. Differential expression and TE analysis were performed on the counts matrix using the DESeq2 (v1.48.2; https://bioconductor.org/packages/release/bioc/html/DESeq2.html) package. Genes with |log_2_fold-change| > 1 and *p* value < 0.05 were considered significantly changed in both RNA-seq and Ribo-seq analyses. For TE differential analysis, genes with |log_2_fold-change of TE| > 1 and *p* value < 0.05 were defined as significantly changed in TE. The complete gene lists for differential expression analysis are provided in Tables S3 and S4. Subsequent analysis was performed using the riboTransVis (v0.0.9) R package.

### Enrichment analysis

GO enrichment analysis was performed using the R package clusterProfiler (v4.12.5). GO terms with a *p*-value < 0.05 were considered significantly enriched.

### Statistical analysis

Statistical analyses were performed using GraphPad Prism software (version 10.6; https://www.graphpad.com). Data are presented as mean ± SD. For comparisons involving two groups, two-tailed Student’s *t* test was used. For comparisons involving three or more groups, one-way ANOVA was used as appropriate, followed by *post hoc* tests (Tukey’s or Dunnett’s test) as indicated in the figure legends. Exact *p* values are provided in the corresponding figures. A *p* value < 0.05 was considered statistically significant.

## Data availability

The Ribo-seq and RNA-seq data generated in this study have been deposited in the National Center for Biotechnology Information Gene Expression Omnibus (GEO) and are accessible through the GEO Series accession number GSE318184 (token password: qbmhwkcybdkfhel).

## Supporting information

This article contains supporting information.

## Conflict of interest

The authors declare that they have no conflicts of interest with the contents of this article.
