# Supplementary material for: mRNA translational dynamics mediates totipotent-like reprogramming
Source: J Biol Chem. 2026 Aug 19;302(10):113464. doi: 10.1016/j.jbc.2026.113464 (PMC13584033; doi:10.1016/j.jbc.2026.113464)
Supplement: Supplementary file 1 [file mmc1.pdf]

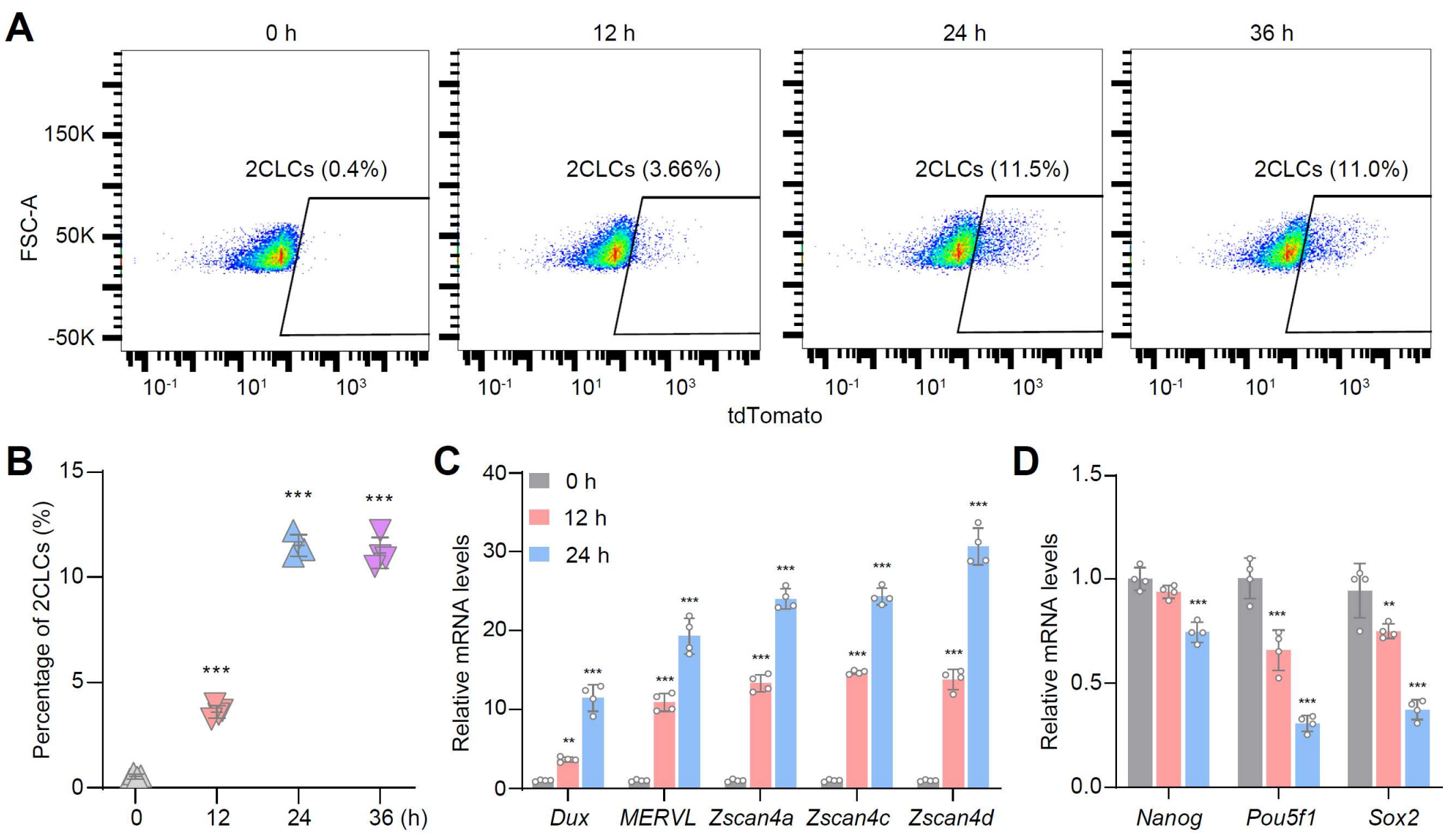

Figure S1. Validation of the totipotent-like reprogramming model.

(A) Flow cytometry analysis showing the percentage of 2C-tdTomato-positive cells at different timepoints during totipotent-like reprogramming. (B) Quantification of the percentage of 2C-tdTomato-positive cells. Data are presented as mean±S.D. from four independent biological replicates (n = 4); $^{***}p < 0.0001$, one-way ANOVA with Dunnett's multiple comparisons test. (C) Detection of totipotency-associated gene expression by RT-qPCR during totipotent-like reprogramming. (D) Detection of pluripotency-associated gene expression by RT-qPCR during totipotent-like reprogramming. All data in (C) and (D) are presented as mean±S.D. from independent biological replicates (n = 4); $^{**}p < 0.01$, $^{***}p < 0.0001$, one-way ANOVA with Dunnett's multiple comparisons test.

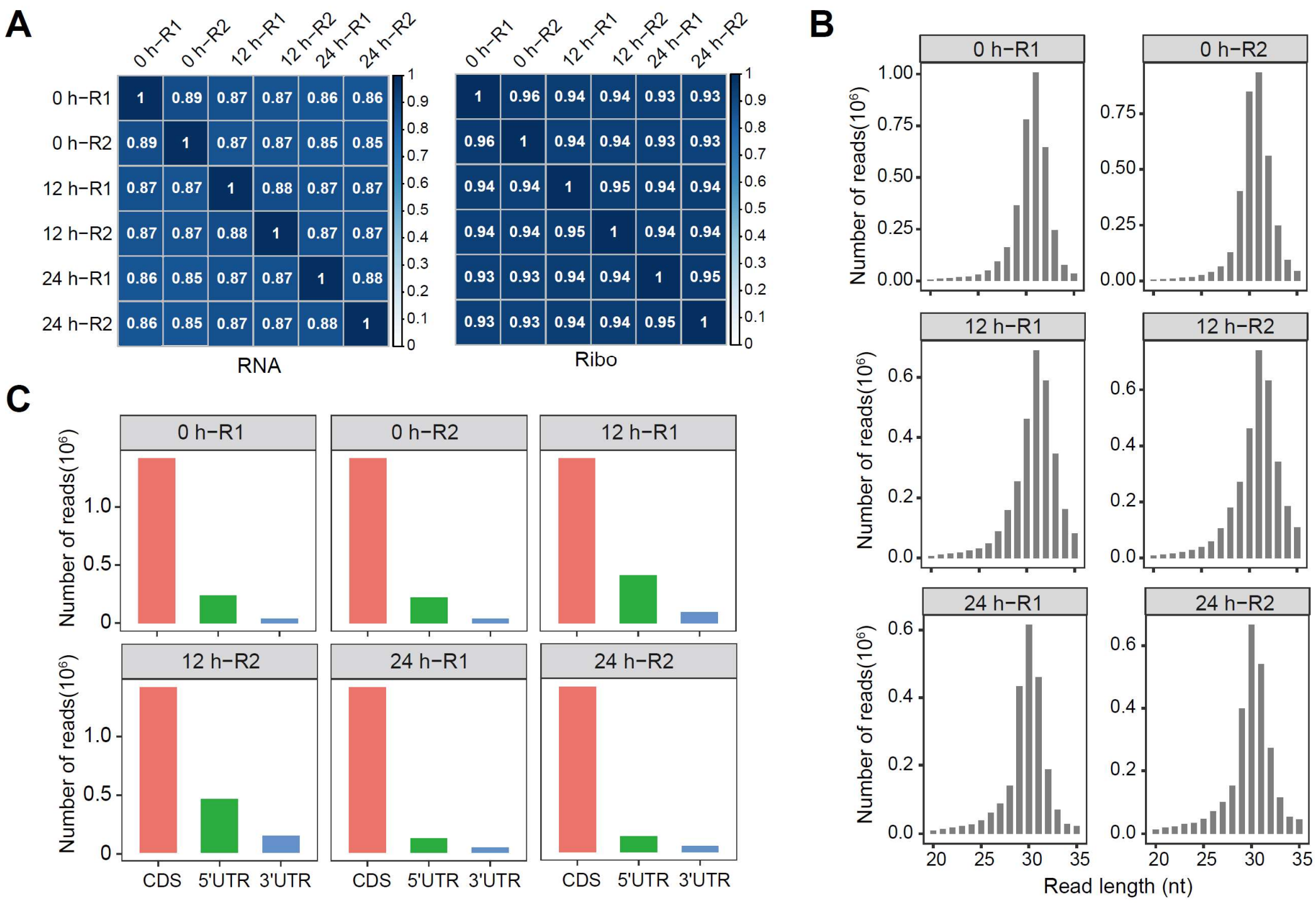

Figure S2. The quality analysis of RNA-seq and Ribo-seq datasets.

(A) Correlation analysis between transcriptomic (RNA-seq) and translatomic (Ribo-seq) data across reprogramming time points (0, 12, and 24 h). (B) Distribution of ribosome-protected fragment (RPF) lengths. A characteristic size distribution peaking at ~30 nucleotides is observed in all samples. (C) Genomic annotation of ribosome footprints. The majority of RPFs map to coding sequences (CDSs), with a smaller fraction located in the 5′ and 3′ untranslated regions (UTRs).

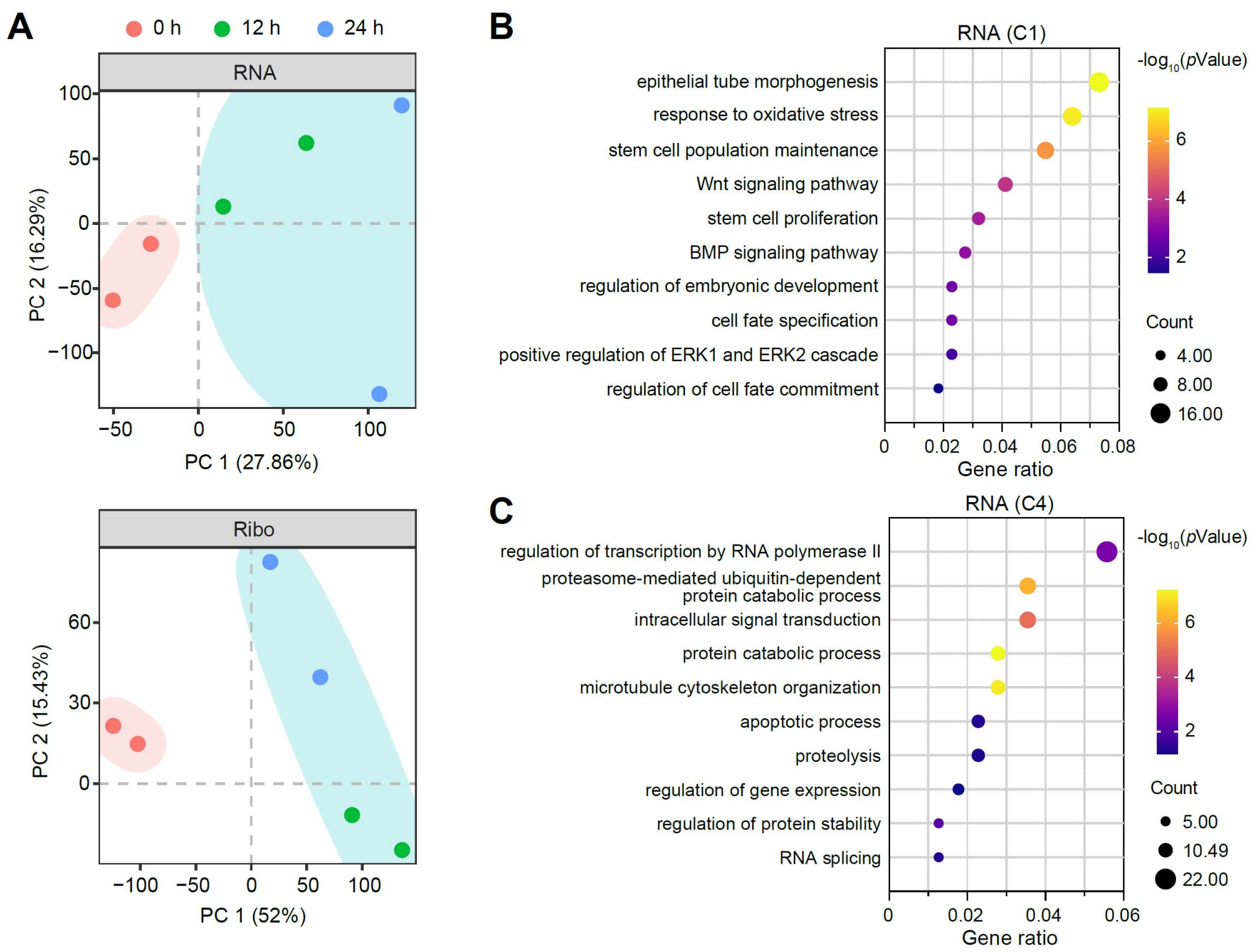

Figure S3. The dynamic changes of RNA abundance and ribosome density during totipotent-like reprogramming.

(A) Principal component analysis (PCA) plot of RNA-seq and Ribo-seq samples. (B) Gene Ontology enrichment analysis for biological processes associated with genes of cluster 1 shown in Fig 1C. (C) Gene Ontology enrichment analysis for biological processes associated with genes of cluster 4 shown in Fig 1C.

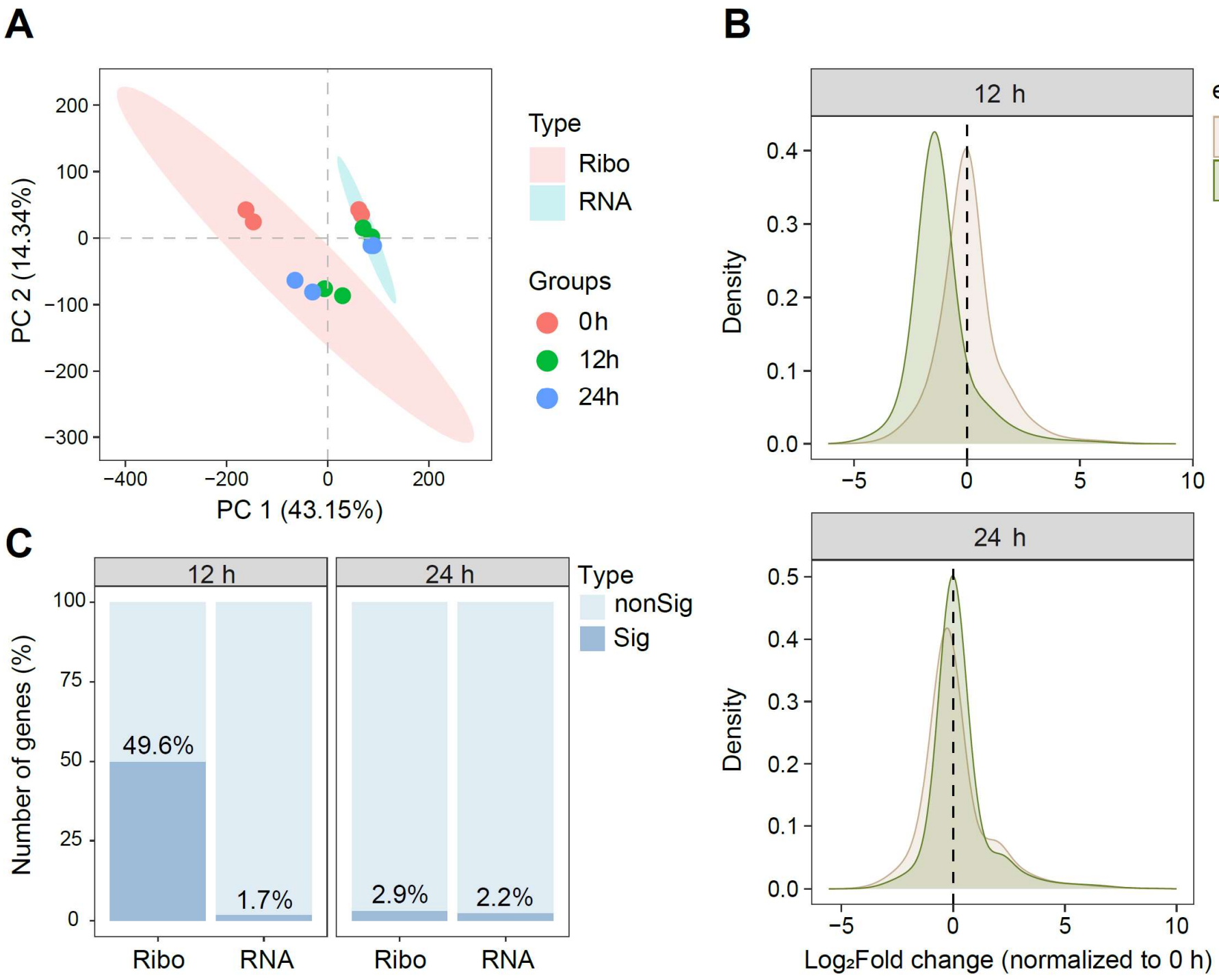

Figure S4. The translatome undergoes more extensive remodeling than the transcriptome during totipotent-like reprogramming

(A) Principal component analysis (PCA) of RNA-seq and Ribo-seq samples at 0 h, 12 h, and 24 h. (B) Density distribution of $\log_2$fold change (FC) for RNA and RPF at 12 h and 24 h (relative to 0 h). (C) Proportion of genes with significant changes ($|\log_2FC| > 1$, p value < 0.05) in RNA-seq and Ribo-seq at 12 h and 24 h.

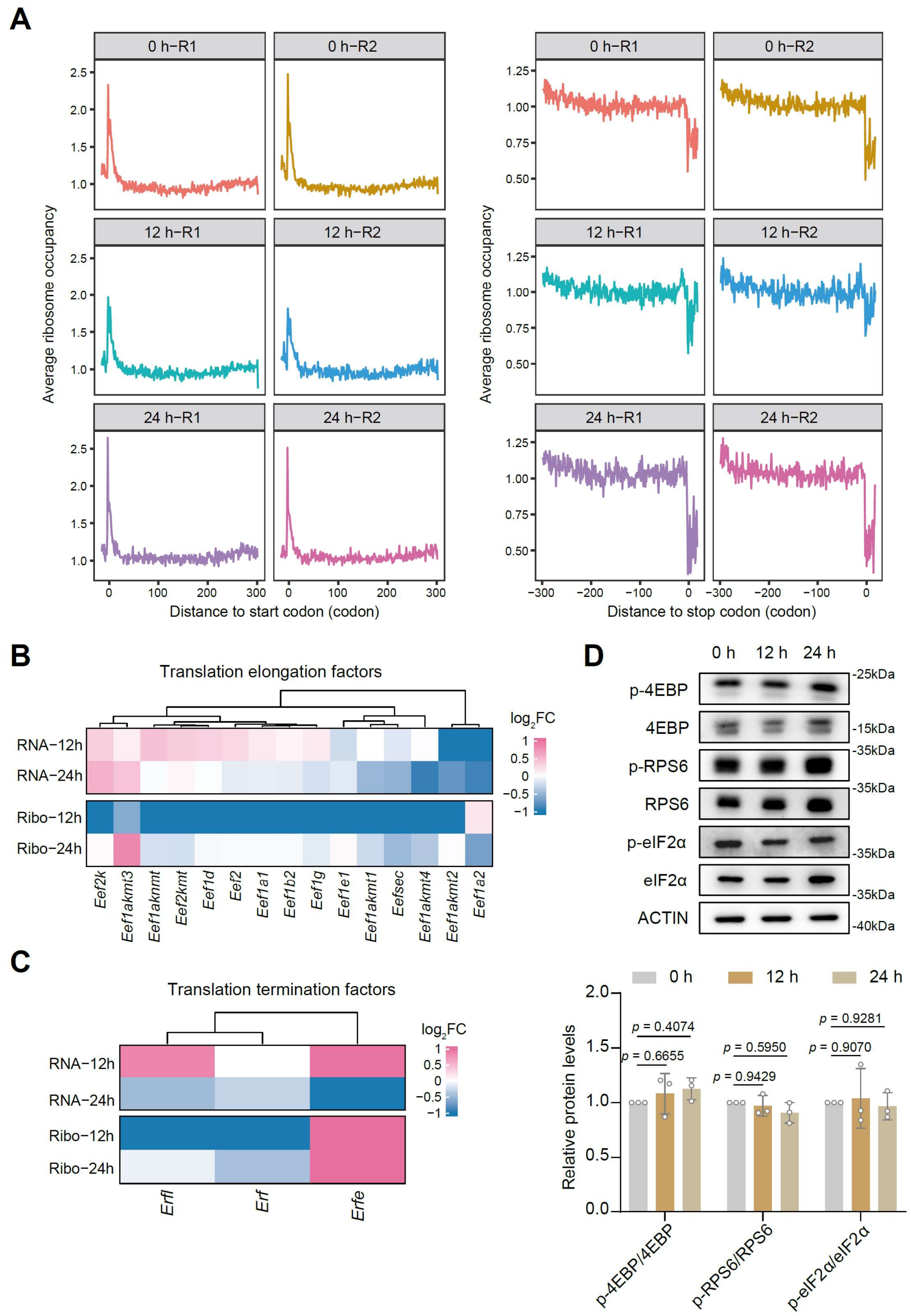

Figure S5. Analysis of translation initiation and termination dynamics during totipotent-like reprogramming.

(A) Metagene analysis of average ribosome density surrounding the translation initiation site (TIS, left) and the translation termination site (TTS, right) of all annotated transcripts during reprogramming. (B) Heatmap of mRNA abundances, and ribosome densities for translation elongation factors across different timepoints during reprogramming. (C) Heatmap of mRNA abundances, and ribosome densities for

translation termination factors across different timepoints during reprogramming. (D) Immunoblot analysis of mTOR pathway-related and ISR pathway-related regulators in *MERVL*-tdTomato reporter cells lacking the Dox-inducible *Dux* system, following Dox treatment alone (2 μg/mL) at 0 h, 12 h, and 24 h. Top: Representative blots showing the phosphorylation and total protein levels of 4EBP1, RPS6, and eIF2α. Bottom: Quantification of p-4EBP/4EBP, p-RPS6/RPS6, and p-eIF2α/eIF2α ratios, normalized to the 0 h control. Data are presented as mean±S.D. from independent biological replicates (n = 3); exact *p* values were included, one-way ANOVA with Dunnett's multiple comparisons test.

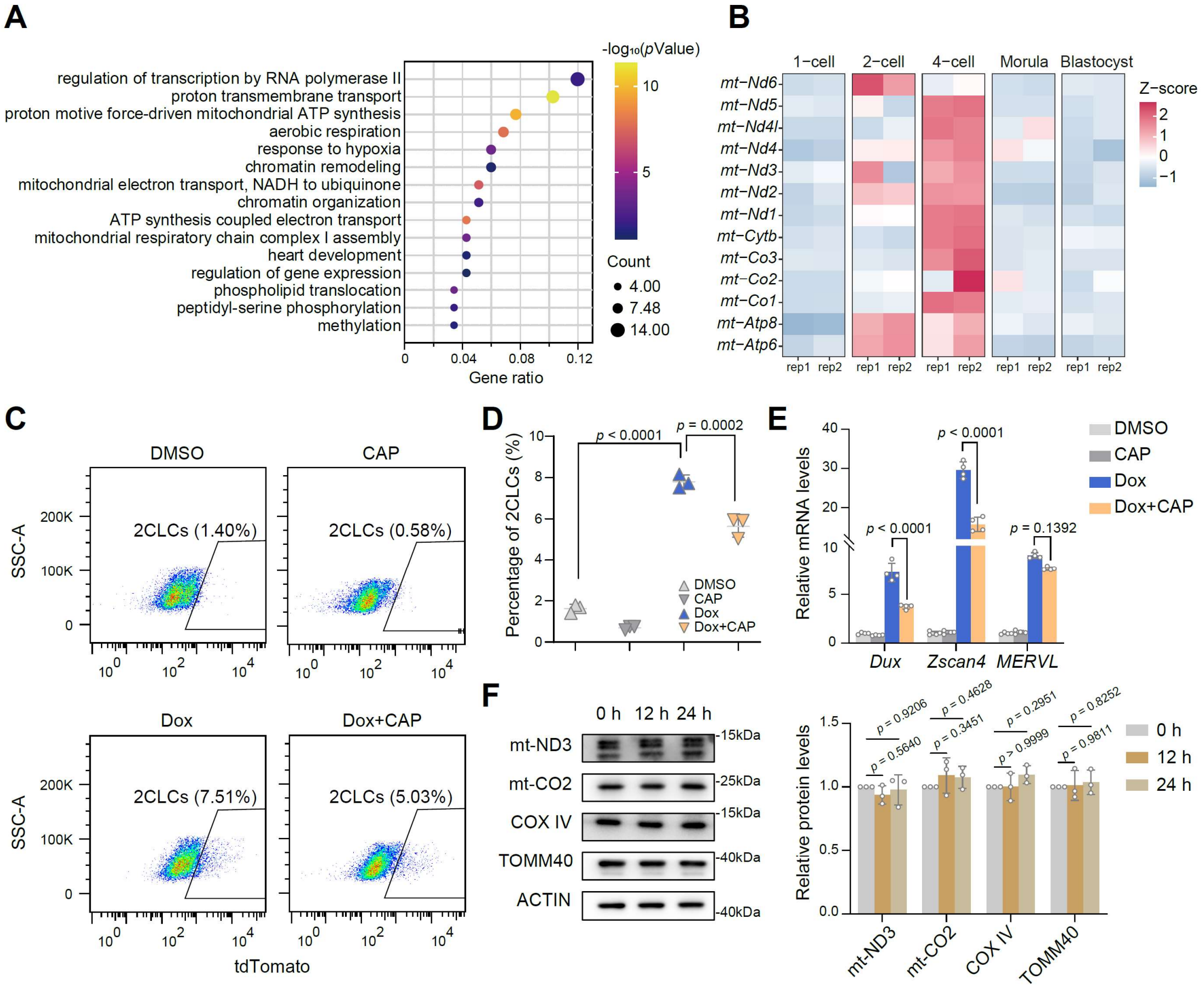

Figure S6. Mitochondrial translation is selectively upregulated to promote totipotent-like reprogramming.

(A) Gene Ontology enrichment analysis for biological processes associated with genes exhibiting significantly upregulated translational efficiency at 24 h during totipotent-like reprogramming. (B) Heatmap showing the translation efficiency (TE) of the 13 mitochondrial-encoded genes across mouse early embryonic developmental stages (1-cell, 2-cell, 4-cell, Morula, and Blastocyst) based on a published dataset (GSE169632). (C) Flow cytometry analysis showing the percentage of 2C-tdTomato-positive cells in the absence or presence of CAP (100 μg/mL, 24 h) in 2i/LIF conditions. (D) Quantification of the percentage of 2C-tdTomato-positive cells in the absence or presence of CAP in 2i/LIF conditions. Data are presented as mean±S.D. from three independent biological replicates (n = 3); exact *p* values were included, one-way ANOVA with Tukey's multiple comparisons test. (E) Detection of 2C gene (*Dux*,

Zscan4, *MERVL*) expression by RT-qPCR in the absence or presence of CAP in 2i/LIF conditions. Data are presented as mean±S.D. from four independent biological replicates (n = 4); exact *p* values were included, one-way ANOVA with Tukey's multiple comparisons test. (F) Immunoblot analysis of mitochondrial protein levels in *MERVL*-tdTomato reporter cells lacking the Dox-inducible *Dux* system, following Dox treatment alone (2 μg/mL) at 0 h, 12 h, and 24 h. Left: Representative blots showing the protein levels of mitochondrial-encoded proteins (mt-CO2, mt-ND3) and nuclear-encoded mitochondrial proteins (COX IV, TOMM40). ACTIN is shown as a loading control. Right: Quantification of the protein levels, normalized to the 0 h control. Data are presented as mean±S.D. from three independent biological replicates (n = 3); exact *p* values were included, one-way ANOVA with Dunnett's multiple comparisons test.

Table S1. Primers for RT-qPCR used in this study.

Gene	Forward primer (5'-3')	Reverse primer (5'-3')
β-Actin	TTGCTGACAGGATGCAGAAG	ACTCCTGCTTGCTGATCCACAT
Dux[*]	AAAGGAAGAGCATGTGCCAGC	GCAGTAAGCTGTCCTGGGAAC
Zscan4a	CCTGCCATTCCCTCTGACAT	ATTATTCCACTTTGAACGTCGTT
Zscan4c	TCTTTCTGGTTGGCAGCTTT	GCCAGGCTTCTGTCAAGAAC
Zscan4d	AAGAGGTGAGGTGGAGGAGT	AAGGTCTTTTGCTGGTGCCT
MERVL	ATCTCCTGGCACCTGGTATG	AGAAGAAGGCATTTGCCAGA
Nanog	CAGCATCCATTGCAGCTATCC	CTGCCCCACATGGAAAGG
Pou5f1	TCGGACCAGGCTCAGAGGTA	ATCCCTCCGCAGAACTCGTA
Sox2	AAACCAAGACGCTCATGAAGAAG	CGCTCGCCATGCTGCTGTTC

*Primers specifically target the endogenous *Dux* transcript and do not recognize the codon-optimized transgenic syn*Dux* sequence.
